# The Direct and Structure Effect of Income on Nutrition Demand of Chinese Rural Residents

**DOI:** 10.3390/ijerph192013388

**Published:** 2022-10-17

**Authors:** Qiyan Zeng, Zhipeng He, Yuting Wang

**Affiliations:** 1 School of Economics and Management, Zhejiang A&F University, Hangzhou 311300, China; 2 Research Academy for Rural Revitalization of Zhejiang Province, Zhejiang A&F University, Hangzhou 311300, China; 3 Institute of Ecological Civilization, Zhejiang A&F University, Hangzhou 311300, China; 4 School of Economics and Management, China Agricultural University, Beijing 100083, China; 5 Beijing Food Safety Policy and Strategy Research Base, China Agricultural University, Beijing 100083, China

**Keywords:** nutrition transition, income, calorie intake, nutrition structure, rural residents

## Abstract

Although a significant body of literature has analyzed the effect of income-mediated policies on nutrition, research on how income affect nutrition consumption is scant. This paper contributes to the literature by decomposing the overall income effect on rural residents’ calorie intake into the direct income effect and the structure effect by building a simple theoretical model and conducting related empirical research with an instrumental variable (IV) approach. Using nationally representative data from China, we find that the structure effect of income, represented by fat share growth induced by income, occupies a considerable proportion (38.03%) of overall income effect. Additionally, we provide evidence of an asymmetric distributional effect of income on calorie intake. In particular, the structure effect of income substantially accounts for a larger proportion in the higher quantiles of the calorie intake distribution. Our findings help better evaluate the effectiveness of the income-mediated policies from quantity and structure perspectives in a comprehensive framework.

## 1. Introduction

Understanding nutrition demand is becoming an increasingly important issue for food security in the face of threats from decreasing agricultural resources and ecological security. Notably, issues of inadequate calorie intake persist in many rural areas of developing countries, whereas nutrition transition has become a new notable issue, as the pace of a nutritional shift from a traditional low-fat diet to a high-fat and high-calorie diet has been accelerating [1,2]. Such a double nutrition burden and its related health consequences have raised widespread concern, especially considering the relatively poor public hygiene facilities and health services in rural areas [3]. Income-mediated policies are the ones most often used to combat calorie-poverty and improve nutrition by governments and non-profit organizations, because income is a basic and core constraint on food purchasing power [4,5]. Therefore, detecting whether and how rural residents’ nutrition changes with income has some far-reaching policy implications either at the micro or macro level [6].

In the literature, the effects of income on nutrition demand have attracted a lot of concern over the past three decades. Numerous studies have focused on calorie intake, as sufficient calorie intake is a fundamental need for human health and productivity. Generally, to what extent calorie demand responds to income is still controversial in the current literature. A selection of studies confirm the positive calorie–income relationship, especially in developing countries, which implies that a rise in income can result in a substantial improvement in malnutrition. For example, Gao et al. (2020) found that an increase in household income will positively influence calorie demand in China [7]. Van den Broeck et al. (2021) confirmed that income growth in rural Tanzania improves nutrition, both in terms of calorie quantity and food quality [8]. Other studies such as Ogundari et al. (2016) and Colen et al. (2018) for sub-Saharan Africa and Africa [9,10], Thi et al. (2018) for Vietnam [11], Bhuyan et al. (2020) for India [12], Shabnam et al. (2021) for Pakistan [13], and Ali et al. (2018) for Nepal [14] have also found a positive relationship between income and calorie demand. However, another strand of literature suggests that calorie demand is income-inelastic, which indicates that households are able to smooth calorie consumption even if income changes. For instance, through the systematic review of the recent literature in nutrition economics, Finaret and Masters (2019) concluded that households will improve the food quality rather than increase the quantity of calories when incomes rise [15], similar to the study of Irz (2010) for Finland [16] and Skoufas et al. (2011) for Indonesia [17]. Obviously, there is still ongoing debate regarding the effect of income on calorie intake. Besides, with the prevalence of nutrition transition, some researchers have started to analyze the effect of income on the nutrition structure, especially represented by macronutrient changes. Macronutrients form the main bulk of food, which mainly consist of carbohydrates, proteins, and fats [1]. A general finding is that as income rises, fat becomes a more important source of energy in adults’ diet, while the proportion of energy derived from carbohydrates quickly declines correspondingly [1,10,18,19,20,21]. For example, Ren et al. (2019) found that wealthier adults are more likely to consume more fat and protein and less carbohydrates in China [1]. It is an important mechanism of income impact on the adult health outcomes of overweight. In addition, the role of income growth in improving dietary diversity, nutritional quality, and micronutrient intake has also attracted considerable interest among many scholars [8,22,23].

Although a significant body of literature has been devoted to the topic, calorie intake and nutrition structure changes are treated as two separate outcomes of income, which fragment our understanding of how income-mediated policies play a role in combatting calorie poverty. That is, they do not further analyze the mechanism of income on calorie consumption, which is essential in the context of accelerated nutrition transition. Broadly speaking, income can influence calorie intake in two ways: by directly affecting the quantity of calorie intake with nutrition structure remaining unchanged, and by affecting nutrition structure. We define the former as the direct effect and the latter as the structure effect, and the sum of them is the overall effect of income on calorie consumption. A “healthy” calorie consumption includes sufficient calories as well as appropriate nutrition structure [4]. Hence, clarification of the direct effect and the structure effect of income is important when we explore whether income-mediated policies can contribute to “healthy” calorie consumption. 

The overall goal of this study is to explore the direct and structure effect of income on rural residents’ calorie consumption. Specifically, first, we decomposed the overall income effect on calorie intake into the direct effect and the structure effect by building a simple theoretical model; second, we conducted related empirical research with an instrumental variable (IV) approach and further evaluated how these effects change across the distribution of calorie intake. The present paper uses data from China, the largest developing country where significant income growth and rapid nutrition transition have been taking place simultaneously in rural areas since the 21st century [22]. Taking China as a research object is expected to be representative, and our findings may, to a certain degree, have some policy implications for other developing countries. 

We try to extend the previous research in the following two ways. First, this paper provides new insight on the mechanism of income affecting calorie consumption, which sheds light on both direct and structure effects of income rather than focusing on one. It helps us better evaluate the effectiveness of income-mediated policies from quantity and structure perspectives in a complete framework. Second, we apply an IV approach to examine whether the estimated impact of income on calorie consumption can be interpreted as causal, and further employ an instrumental variable quantile regression (IVQR) framework to study the potential asymmetry in the causal influence of income on the distribution of calorie intake.

The remainder of this paper is organized as follows: Section 2 introduces the data and methods, Section 3 presents the empirical results, and finally, the paper is discussed and concluded in Section 4.

## 2. Materials and Methods

### 2.1. Data

The data for this study were drawn from the China Health and Nutrition Survey (CHNS), an ongoing open cohort, international collaborative project between the Carolina Population Center at the University of North Carolina at Chapel Hill and the National Institute for Nutrition and Health at the Chinese Center for Disease Control and Prevention. The CHNS is a widely recognized long-term survey that sheds light on nutrition and health issues for the Chinese population. The survey began in 1989 and there have been ten rounds of surveys to date, of which the latest was in 2015. A multistage cluster random sampling method was used to derive the original sample, which covers about 4000–5000 households every year. Related information is collected by questionnaire surveys on the individual, household, and community levels over a 7-day period. The CHNS contains key variable information we need, such as food consumption and income, which fully supports our analysis. 

Given the new phase of residents’ food consumption since the 21st century, our analysis includes adults aged 18 and above in the 2000–2011 survey. Unfortunately, data for food consumption of the 2015 survey are unpublished and thus not included in the present paper. After dropping the observations with missing values or outliers (calorie intake is higher than 5000 kcal/d or lower than 700 kcal/d), we have a final sample of 30,344 adults, and the distribution among the years 2000, 2004, 2006, 2009, and 2011 is 5252 (17.31%), 5920 (19.51%), 5884 (19.39%), 6236 (20.55%), and 7052 (23.24%).

### 2.2. Theoretical Framework

We firstly constructed a simple analysis framework to decompose the overall effect of income on calorie consumption into the direct effect and the structure effect, which lay the foundation for the following empirical strategy. Specifically, we assume individuals derive utility from two composite goods: calorie producing goods, *C*, and non-calorie producing goods, *Q* [24]. The utility function can be then defined as:

*U* = *U*(*C*,*Q*) (1)

Given an income budget, we have P*C + Q ≤ I. *P* is the relative food price and *I* is the income. Calorie intake *C* mainly derives from macronutrients in food, and it is also influenced by the macronutrient structure [25,26]. Several factors such as caloric density, satiety properties, and post-absorptive processing of different macronutrients can contribute to the difference in energy intake level [27]. Particularly, it is emphasized that high-fat diets can induce over-consumption and weight gain through their low satiety properties and high caloric density [28,29]. Hence, calorie intake could be assumed to be:

*C* = *C*(*S*,*Z*) (2)

where *S* is nutrition structure and *Z* denotes other demographic and socio-economic characteristics affecting calorie consumption.

Nutrition structure is influenced by income changes [1,4,10], that is:

*S* = *S*(*I*) (3)

Combined with Equations (1)–(3) and the budget constraint, individuals can make an optimal calorie consumption decision C* by maximizing the utility function:

C* = *C*(*I*, *S*(*I*), *P*, *Z*) (4)

To obtain the effect of income on calorie consumption, we take the partial derivative with respect to *I* for Equation (4):

(5) $$\frac{dC}{dI}\left|\mathrm{overall}\;\mathrm{effect}=\frac{\partial C}{\partial I}\right|\mathrm{direct}\;\mathrm{effect}+\left(\frac{\partial C}{\partial S}*\frac{\partial S}{\partial I}\right)|\mathrm{structure}\;\mathrm{effect}$$

where *dC/dI* is the overall effect of individuals’ income on calorie intake, which can be decomposed into the direct income effect (the first term on the right side *∂C/∂I*) and the structure effect (the second term on the right side *∂C/∂S* ∗ *∂S/∂I*). Specifically, the direct income effect measures calorie quantity expansion with a fixed nutrition structure, while the structure effect of income measures calorie changes driven by nutrition structure transition. In the next section, we introduce the detailed empirical strategy based on the theoretical model.

### 2.3. Empirical Strategy

We suppose *C(I, S(I), P, Z)* and *S(I)* to be a linear function based on the framework of Aromolaran (2004) [24], Zhong et al. (2012) [30], Nie and Sousa-Poza (2016) [5], and Van den Broeck et al. (2021) [8], and then we have the following specifications:

(6) $$\ln C_{i}=\gamma_{0}+\gamma_{1}\ln I_{i}+\gamma_{2}Z_{i}+\varepsilon_{i}$$

(7) $$\ln C_{i}=\alpha_{0}+\alpha_{1}\ln I_{i}+\alpha_{2}S_{i}+\alpha_{3}Z_{i}+\delta_{i}$$

(8) $$S_{i}=\beta_{0}+\beta_{1}\ln I_{i}+\beta_{2}Z_{i}+e_{i}$$

where $\ln C_{i}$ is log-transformed individual average daily calorie intake, and $\ln I_{i}$ denotes the log-transformed individual income. $S_{i}$ is nutrition structure, represented by the share of calories obtained from fat. $Z_{i}$ denotes a matrix of control variables.

$\gamma_{1}$, $\alpha_{1}$, $\alpha_{2}$, and $\beta_{1}$ are the parameters of interest. $\gamma_{1}$ measures the overall effect of income on calorie intake. $\alpha_{1}$ measures the direct effect of income as the structure of calories controlled in Equation (7), whereas $\left(\alpha_{2}*\beta_{1}\right)$ measures the structure effect of income, that is:

(9) $$\frac{dC}{dI}\left|\mathrm{overall}\;\mathrm{effect}=\gamma_{1}=\frac{\partial C}{\partial I}\right|\mathrm{direct}\;\mathrm{effect}+\left(\frac{\partial C}{\partial S}*\frac{\partial S}{\partial I}\right)|\mathrm{structure}\;\mathrm{effect}=\alpha_{1}+\left(\alpha_{2}*\beta_{1}\right)$$

However, when analyzing the relationship between income and calorie or nutrition intake, we should take the endogeneity issue into account, especially considering the omitted variable bias and the simultaneity bias. For the former, some unobserved individual heterogeneity (e.g., personality factors, work ability) may affect both calorie demand and income simultaneously. For the latter, if labor productivity, an important determinant of income, depends on nutrition intake, individual income cannot be treated as exogenous in the nutrient demand analysis [21]. In this case, the ordinary least squares (OLS) estimation may be biased. Therefore, instrumental variables are widely employed to tackle the bias, and a variety of instruments for income have been adopted in numerous studies, such as the number of durable assets [5,31], non-food expenditure [32], characteristics of working people [21], and rainfall [33]. 

Based on the current literature and the availability of data in the CHNS, we employ the number of household durable assets as the instrumental variables for income. Specifically, household durables include TV, refrigerator, air-conditioner, camera, VCR, washing machine, fan, etc. The stock of household durables is an efficient proxy of wealth, and it is informative for household income; besides, durable assets reflect saving from prior earnings, and thus, current labor productivity is not directly associated with the durables in a static setting [5,34]. Therefore, as it is emphasized by Skoufias et al. (2009) [31], Nie and Sousa-Poza (2016) [5], and Doan (2014) [35], the number of durable assets in the family are expected to be valid instrumental variables for income. 

Specifically, we propose to estimate the following two-stage least squares (2SLS) model to measure the effect of income on calorie intake instead of Equation (6):

(10) $$\ln I_{i}=\rho_{0}+\rho_{1}A_{i}+\rho_{2}Z_{i}+\sigma_{i}$$

(11) $$\ln C_{i}=\gamma_{0}^{\prime }+\gamma_{1}^{\prime }\hat{\ln I_{i}}+\gamma_{2}^{\prime }Z_{i}+\varepsilon_{i}^{\prime }$$

where the first-stage model in Equation (10) is the income determination equation, and $A_{i}$ denotes the instrumental variable (household durable assets). The second-stage regression model in Equation (11) explains observed variation in calorie intake using predicted value $\hat{\ln I_{i}}$ from Equation (10). $\gamma_{1}^{\prime }$ measures the overall effect of income on calorie intake. Similarly, we can derive $\alpha_{1}^{\prime }$, $\alpha_{2}^{\prime }$, and $\beta_{1}^{\prime }$ by estimating the 2SLS model as follows:

(12) $$\ln C_{i}=\alpha_{0}^{\prime }+\alpha_{1}^{\prime }\hat{\ln I_{i}}+\alpha_{2}^{\prime }S_{i}+\alpha_{3}^{\prime }Z_{i}+\delta_{i}^{\prime }$$

(13) $$S_{i}=\beta_{0}^{\prime }+\beta_{1}^{\prime }\hat{\ln I_{i}}+\beta_{2}^{\prime }Z_{i}+e_{i}^{\prime }$$

In addition, since the effect of income may differ across the calorie distribution, and at the same time, income itself may be endogenous, we further utilize the instrumental variable quantile regression approach proposed by Chernozhukov and Hansen (2005, 2008) to analyze the effect of income on the distribution of calorie intake [36,37].

### 2.4. Measurement of Variables

#### 2.4.1. Calories and Nutrition Structure

The CHNS provides detailed individual food intake information through in-person interviewer-administered 24 h recalls that are conducted by trained staff over 3 consecutive days, including 2 working days and 1 weekend day. Total energy is summed from individual reported foods and from weighted household condiments, mainly cooking oil. Household condiment consumption is calculated by examining changes in condiment levels from the beginning to the end of each day during the surveyed 3 days and is then distributed among members by the proportion of food intake in households. 

Based on the recorded food items and amounts, the 3-day average calorie intake (kcal) and macronutrient intake (g) are coded according to the China Food Composition Table. Furthermore, considering the prevalent features of nutritional transformation and the worrying outcome of high-fat diet, we mainly focus on the share of calories obtained from fat rather than others as an important indication for the measurement of nutrition structure [21]. That is, the structure effect of income in our paper specifically refers to the change in calorie intake that is driven by the change in the fat share induced by income.

#### 2.4.2. Income

Income is measured by per capita annual household income inflated to 2011, and this definition is consistent with other related studies [5,21,30]. Income is attributable to nine sources in the CHNS: businesses, farming, fishing, gardening, livestock, subsidies, retirement pension, nonretirement earnings, and others. We do not use total individual annual income data, as the data are severely missing and the family is a basic unit in food consumption.

#### 2.4.3. Control Variables

Additional independent variables include demographic and socio-economic characteristics that are supposed to influence calorie intake according to previous studies [5,6,9,21,30]. Age, gender, education level, and marital status are chosen as demographic characteristics for controlling differences among individuals. Socio-economic variables include work-related labor intensity, household size, and food price. For labor intensity, respondents were asked about the activity levels of their occupations, and interviewers categorized them into five activity levels (very light, light, moderate, heavy, and very heavy) based on respondents’ job descriptions and the time spent sitting, standing, walking, and lifting heavy loads during an average work day. Moreover, since data for food prices are unpublished in the CHNS, we use food retail prices taken from the *China Statistical Yearbook* for each of the provinces in the respective years and adjusted by general CPI [30]. In addition, surveyed years and regions are also controlled for the effects of time and geographical differences. Table 1 reports the statistic descriptions of variables.

## 3. Results

### 3.1. The Effect of Income on Calorie Intake

The regression results from the OLS and IV estimation are presented in Table 2. The endogeneity test shows that income is an endogenous variable in both calories and fat intake estimation, as the null hypothesis that income is exogenous is rejected at the conventional level of significance (*p* < 0.01). That is, the results support the necessity of the IV estimation. As expected, we find that the number of durable assets has a significantly positive association with per capita annual household income. Moreover, F-statistics are larger than 10, suggesting that all IV estimation has no weak instrument problem. Therefore, the regression results from the IV estimation are mainly used to analyze the effect of income on calorie and fat intake.

Given the results of the IV estimation presented in Table 2, the coefficients of interest are all positive and statistically significant. The dependent variable in columns (1), (2), (4), and (5) is calorie intake, but columns (2) and (5) additionally control for fat share. The dependent variable in columns (3) and (6) is fat intake. The overall calorie–income elasticity is 0.071, which indicates that a 1% increase in income will boost per capita calorie intake by 0.071%. The parameter for the direct effect of income on calorie intake equals 0.044, and the parameter for the structure effect is 0.027 (0.445 × 0.060 ≈ 0.027). That is, 38.03% of the calorie consumption increase is driven by the fat share increase induced by income growth, while the remaining part is attributed to the direct income effect, that is, calorie quantity expansion with constant fat share. 

Compared to the results of the OLS estimation presented, the coefficients of key variables are in the same direction, whereas the OLS underestimates the overall effect and the structure effect of income on calorie intake due to potential endogeneity. Hence, endogeneity of income is emphasized in numerous studies when we investigate the relationship between income and food consumption [5,21].

Furthermore, demographic and socio-economic characteristics also appear to affect individuals’ calorie intake and fat share. For instance, energy intake decreases with age, education level, and food price, while it increases with household size and body activity level. The proportion of energy from fat is larger for individuals who are male, unmarried, and have a lower activity level. These results are mostly consistent with previous studies.

For the robustness test, we further checked this kind of effect by running subsample regression for each surveyed year. The results, as shown in Table A1, largely support the robustness of the results. Although it presents a decreasing trend, the structure effect of income on calorie demand does play an important role between years.

### 3.2. The Heterogeneous Effects of Income on Calorie Intake

Heterogeneity among people should not be ignored due to potential differences in eating habits, budget constraint, dining facilities, etc. The previous section presents the average effect of income on calorie intake for the population, but this impact may differ at different quantiles of calorie distribution. Hence, we explore the impact of income on the distribution of adults’ calorie consumption in Table 3, which allows us to better understand when, and to what extent, income affects calorie intake.

The IVQR estimates reveal that the mean effect derived from the IV estimates masks considerable heterogeneity. Specifically, the elasticity of income is always significantly positive at quantiles 0.15–0.85, decreasing from 0.108 at the lowest quantile to 0.040 at the 85th quantile. However, the proportion of the structure effect of income shows a general upward trend, increasing from 23.15% to 60.00% between the 15th and 85th quantiles. This indicates that, conditional on other covariates, the structure effect of income accounts for a larger proportion of adults who consume more calories.

We next examine whether the impact of income differs by place of gender and age, which are common and easy to capture in policy design. The heterogeneous results of gender are presented in Table 4. In regard to gender, the results demonstrate that the structure effect of income plays a relatively more important role in females’ calorie consumption increase. The income elasticity of calorie consumption is the same for males and females; however, for females, 38.03% of calorie consumption increase is driven by increasing fat proportion induced by income growth, which is slightly higher than that of males (33.80%).

In regard to age, the heterogeneous results of age are presented in Table 5. From the Table 5, we find that the overall calorie–income elasticity is the largest for middle-aged people, whereas the structure effect of income accounts for the largest share for young people. Specifically, the whole sample is divided into the young group (age < 45), the middle-aged group (45 ≤ age < 65), and the elderly group (age ≥ 65) [38]. According to the results presented in Table 5, the overall calorie–income elasticity is the largest for middle-aged people (0.070), whereas the share of structure effect is the smallest (30%), which indicates that calorie quantity expansion with constant fat share still dominates. More than half of the positive effect of income on calories can be attributed to increasing fat share driven by income growth for young people, which implies that nutrition structure transition plays an important role.

## 4. Discussion

The structure effect of income, represented by fat share growth induced by income, occupies a considerable proportion of overall effect of income on calorie consumption. It implies that income-mediated policies can help to adjust the structure of calorie sources as well as combat insufficient calories. A possible reason for the phenomenon is that diets rich in fats tend to be more flavorful and varied and greatly contribute to eating pleasure, and thus people tend to enjoy more high-fat foods with purchasing power growth [39,40,41]. Our findings echo research focusing on the impact of income on nutritional structure among the Chinese population [1,7,18,21,22]. With income growth, fat becomes an increasingly important source of energy in an adult’s diet. However, we extend this by specifically measuring the proportion of the structure effect in the total income effect. Moreover, high-fat diets induce greater energy intake and weight gain than other macronutrients, and thus excessive fat intake nowadays has raised great concerns [3,42,43]. For example, in our data, the average proportion of energy derived from fat for rural residents in China has increased from 25.64% to 31.68% between the surveyed years, which has already exceeded current recommendations for dietary fat intake in the dietary guidelines for Chinese residents (no more than 30%). It is evident that income growth can help ameliorate issues of inadequate calorie intake, while increasing income will also exacerbate the problem of excessive fat intake. Therefore, how to balance sufficient calorie intake and proper nutrition structure should be considered when income-mediated policies are implemented. A crucial prerequisite is to measure the two effects in a comprehensive and objective way, and our study provides a method to measure the effects in an comprehensive framework, which can also be applied to the evaluation of other policy effects beyond income-mediated policies.

Furthermore, the heterogeneity among the population is worth noting. On one hand, our paper confirms that the structure effect of income substantially accounts for a larger proportion in the higher quantiles of calorie intake distribution. This suggests that an increasing fat share induced by income will be a larger concern with calorie intake rising nowadays. We believe that looking at the calorie intake distribution instead of focusing on average impacts allows us to better understand when, and to what extent, income affects adults’ calorie consumption. On the other hand, our results demonstrate that the structure effect is more prominent for young people and female adults. The gender difference in the structure effect could be due to the fact that females generally prefer high-fat Western-style convenience foods and snacks (e.g., milk tea, cream, butter cookies, etc.) compared to males in China [44,45]. A possible explanation for the difference among adults of different ages is that young people more easily accept a Western-style diet high in fat, which was introduced to China ten years ago, whereas the middle-aged and the elderly are more likely to stick to a traditional Chinese diet [44].

In addition, although we found a positive and statistically significant impact of income on rural residents’ calorie intake, the calorie–income elasticity is small. The findings are consistent with the majority of studies on this topic using data from China [5,7,30,46]. For example, by estimating nutrient–income elasticities separately for urban and rural adults in China, Gao et al. (2020) found that the income elasticities of calories are in the range of 0.059–0.076, which is quite close to the elasticity estimated in the present paper [7]. Nie and Sousa-Poza (2016) also confirmed that the calorie–income elasticities of the Chinese population are generally small, ranging from −0.031 to 0.022, regardless of whether parametric, nonparametric, or semiparametric approaches are used [5]. The low calorie–income elasticity can be partly attributable to the fact that per capita energy intake has generally reached a relatively high level in China, and is thus not very sensitive to income changes overall [30]. Specifically, the average daily calorie intake for adults in our data reached about 2180.60 kcal, which is up to the standard recommended by the World Health Organization. However, this does not mean that income-mediated policies are no longer an effective tool to combat malnutrition. The IVQR estimates also reveal that the elasticity of income with respect to calorie intake continuously increases from the highest quantile to the lowest quantile of the calorie intake distribution, which implies that income is still an important strategy to improve the nutritional status of people with inadequate calorie intake, especially in undeveloped countries or regions.

However, the present study has some limitations. Firstly, due to the availability of the data, our analysis is based on data from 2000 to 2011, which are old and can hardly be representative of the current relationship between income level and nutrition issue. Secondly, due to availability of the data, food retail prices are taken from the *China Statistical Yearbook* for each of the provinces, which may be somewhat biased in each surveyed community. Thirdly, we mainly focus on the fat share as the measurement of nutrition structure, and it would be prudent to expand the results to other nutrition transitions. Therefore, future studies can explore the direct effect and the structure effect of income on various nutrition transitions with updated and richer data. Despite the limitations, our findings can still be suggestive by firstly decomposing the overall effect of income on calorie intake into the direct income effect and the nutrition structure change effect. As such, future studies can evaluate the effect of income-mediated policies in a more comprehensive framework including both quantity and structure. This study can also be applied to the evaluation of other policy effects beyond income-mediated policies.

## 5. Conclusions

Using nationally representative data from China, this study examines the effect of income on rural residents’ calorie consumption by decomposing the overall income effect into the direct income effect and the structure effect. Although a positive and statistically significant impact of income on rural residents’ calorie intake was found, the calorie–income elasticity is small. The structure effect of income, represented by fat share growth induced by income, occupies a considerable proportion (38.03%) of the overall effect of income on calorie consumption. Furthermore, our paper confirms that the structure effect of income substantially accounts for a larger proportion in adults who are young, female, and have a high calorie intake.

Our findings are potentially relevant for policy making that aims at improving nutrition and health. The implementation and assessment of related income-mediated policies should take the structure effect into consideration rather than only concentrate on the calorie quantity. Our paper provides a possible method to simultaneously evaluate the two effects of income-mediated policies in a comprehensive framework, which help predict the calorie consumption expansion in both quality and quantity. In particular, considering the considerable proportion of the structure effect, dietary instruction and education programs should be implemented concomitantly to prevent high-fat diets when income-mediated policies are carried out. Moreover, people who are female, young, and have a high calorie intake should be given more attention due to a more prominent structure effect and higher fat proportion status in these demographic groups.

## Figures and Tables

**Table 1:** Descriptive statistics of variables.

Variables	Definition	Mean	SD
Calories	Average daily energy (kcal/day), in logarithm	7.640	0.315
Fat share	The share of calories obtained from fat	0.282	0.111
Income	Per capita annual household income, in logarithm	8.580	1.078
Age	In years, ≥18	48.451	14.942
Gender	Dummy, male = 0, female = 1	0.517	0.500
Education level	Years of formal education	6.886	4.026
Marital status	Dummy, married = 1, otherwise = 0	0.847	0.360
Activity level	Labor intensity, increasing from 1 to 5	2.909	1.191
Household size	The number of people in the household	3.885	1.624
Food price	The relative food retail prices (1999 = 100)	129.145	22.828
Durable assets	The number of household durable assets	6.741	4.474

**Table 2:** The results from the OLS estimation and IV estimation.

Variables	OLS Estimation			IV Estimation		
	(1) Calories	(2) Calories	(3) Fat Share	(4) Calories	(5) Calories	(6) Fat Share
				Second stage:		
Income	0.024 ***	0.020 ***	0.008 ***	0.071 ***	0.044 ***	0.060 ***
	(0.002)	(0.002)	(0.001)	(0.007)	(0.007)	(0.003)
Fat share		0.462 ***			0.445 ***	
		(0.016)			(0.016)	
Age	−0.002 ***	−0.002 ***	0.0004 ***	−0.002 ***	−0.002 ***	0.0005 ***
	(0.0001)	(0.0001)	(0.0001)	(0.0001)	(0.0001)	(0.0001)
Gender	−0.158 ***	−0.163 ***	0.012 ***	−0.159 ***	−0.163 ***	0.010 ***
	(0.003)	(0.003)	(0.001)	(0.004)	(0.003)	(0.001)
Education	−0.001	−0.002 ***	0.003 ***	−0.003 ***	−0.003 ***	0.0003
	(0.001)	(0.001)	(0.0002)	(0.001)	(0.001)	(0.0003)
Marriage	0.042 ***	0.042 ***	−0.0002	0.035 ***	0.038 ***	−0.007 ***
	(0.005)	(0.005)	(0.002)	(0.005)	(0.005)	(0.002)
Body activity	0.045 ***	0.053 ***	−0.016 ***	0.048 ***	0.054 ***	−0.013 ***
	(0.002)	(0.002)	(0.001)	(0.002)	(0.002)	(0.001)
Household size	−0.001	0.002 *	−0.006 ***	0.004 ***	0.005 ***	−0.001 *
	(0.0004)	(0.001)	(0.0004)	(0.001)	(0.001)	(0.0004)
Food price	−0.004 ***	−0.005 ***	0.0003 *	−0.004 ***	−0.004 ***	0.001 ***
	(0.0004)	(0.0004)	(0.0001)	(0.0004)	(0.0004)	(0.0002)
Region and year variables	control	control	control	control	control	control
				First stage		
Durable assets				0.074 ***	0.072 ***	0.074 ***
				(0.002)	(0.002)	(0.002)
R-squared	0.153	0.177	0.125	/	/	/
Cragg–Donald Wald F statistic				2319.912	2036.453	2319.912
Endogeneity test *p*-value				0.0000	0.0002	0.0000
Share of structure effects	16.67%			38.03%		
Observations	30,344	30,344	30,344	30,344	30,344	30,344

Note: * *p* < 10%, ** *p* < 5%, *** *p* < 1%. The standard deviation is shown in brackets.

**Table 3:** IVQR estimates of the effect of income on calorie consumption.

Variables	Calorie Intake									
	0.15		0.25		0.50		0.75		0.85	
Income	0.108 *** (0.001)	0.083 *** (0.001)	0.093 *** (0.001)	0.059 *** (0.001)	0.078 *** (0.001)	0.051 *** (0.001)	0.059 *** (0.001)	0.029 *** (0.001)	0.040 *** (0.001)	0.016 *** (0.001)
Fat share		0.521 *** (0.025)		0.521 *** (0.022)		0.450 *** (0.020)		0.429 *** (0.022)		0.392 *** (0.025)
Control variables	Yes	Yes	Yes	Yes	Yes	Yes	Yes	Yes	Yes	Yes
Structure effects share	23.15%		36.56%		34.62%		50.85%		60.00%	
Observations	30,344	30,344	30,344	30,344	30,344	30,344	30,344	30,344	30,344	30,344

Note: * *p* < 10%, ** *p* < 5%, *** *p* < 1%. The standard deviation is shown in brackets. Controlled variables include demographic and socio-economic characteristics, survey year, and geographic variables, as shown in Table 1. Table 4 to Table 5 are also consistent with this note.

**Table 4:** Gender gap in the effect of income on calorie consumption.

	Male			Female		
	(1) Calories	(2) Calories	(3) Fat Share	(4) Calories	(5) Calories	(6) Fat Share
Income	0.071 ***	0.047 ***	0.062 ***	0.071 ***	0.044 ***	0.055 ***
	(0.009)	(0.010)	(0.004)	(0.009)	(0.009)	(0.004)
Fat share		0.388 ***			0.496 ***	
		(0.024)			(0.022)	
Controlled variables	Yes	Yes	Yes	Yes	Yes	Yes
Cragg–Donald Wald F statistic	1055.255	992.690	985.336	1166.983	1103.715	1166.983
Endogeneity test *p*-value	0.0000	0.0072	0.0000	0.0000	0.0045	0.0000
Share of structure effect	33.80%			38.03%		
Observations	14,660	14,660	14,660	15,684	15,684	15,684

Note: * *p* < 10%, ** *p* < 5%, *** *p* < 1%. The standard deviation is shown in brackets. For simplicity, results in the first stage of the IV estimation are omitted in Table 4 and Table 5 as they are similar to those in Table 2.

**Table 5:** The effect of income on calorie consumption for different age groups.

	Young People			Middle-Aged People			Elderly People		
	(1) Calories	(2) Calories	(3) Fat Share	(4) Calories	(5) Calories	(6) Fat Share	(7) Calories	(8) Calories	(9) Fat Share
Income	0.061 ***	0.029 **	0.065 ***	0.070 ***	0.049 ***	0.054 ***	0.064 ***	0.040 ***	0.052 ***
	(0.012)	(0.012)	(0.005)	(0.010)	(0.010)	(0.004)	(0.016)	(0.016)	(0.006)
Fat share		0.486 ***			0.399 ***			0.468 ***	
		(0.027)			(0.024)			(0.042)	
Controlled variables	Yes	Yes	Yes	Yes	Yes	Yes	Yes	Yes	Yes
Cragg–Donald Wald F statistic	675.585	614.650	675.585	999.771	967.825	999.771	420.487	410.243	420.487
Endogeneity test *p*-value	0.0005	0.2333	0.0000	0.0000	0.0066	0.0000	0.0005	0.0437	0.0000
Share of structure effect	52.46%			30.00%			37.50%		
Observations	12,293	12,293	12,293	13,584	13,584	13,584	4467	4467	4467

Note: * *p* < 10%, ** *p* < 5%, *** *p* < 1%. The standard deviation is shown in brackets.

## Data Availability

The datasets used and/or analyzed during the current study are available from the corresponding author on reasonable request.

## Appendix A

**Table A1:** The effect of income on calorie consumption among years.

	Dependent Variables		
Year: 2000	Calories	Calories	Fat share
Income	0.078 *** (0.015)	0.028 * (0.015)	0.082 *** (0.006)
Fat share		0.622 *** (0.039)	
Observations	5252		
Year: 2004	Calories	Calories	Fat share
Income	0.100 *** (0.016)	0.054 *** (0.016)	0.073 *** (0.006)
Fat share		0.622 *** (0.038)	
Observations	5920		
Year: 2006	Calories	Calories	Fat share
Income	0.046 *** (0.015)	0.024 (0.015)	0.058 *** (0.006)
Fat share		0.378 *** (0.037)	
Observations	5884		
Year: 2009	Calories	Calories	Fat share
Income	0.066 *** (0.015)	0.036 *** (0.015)	0.046 *** (0.005)
Fat share		0.667 *** (0.038)	
Observations	6236		
Year: 2011	Calories	Calories	Fat share
Income	0.055 *** (0.013)	0.048 *** (0.014)	0.051 *** (0.005)
Fat share		0.139 *** (0.033)	
Observations	7052		

Note: * *p* < 10%, ** *p* < 5%, *** *p* < 1%. The standard deviation is shown in brackets.
